# Differential Ability of Bovine Antimicrobial Cathelicidins to Mediate Nucleic Acid Sensing by Epithelial Cells

**DOI:** 10.3389/fimmu.2017.00059

**Published:** 2017-02-01

**Authors:** Arnaud Baumann, Mirjam Susanna Kiener, Brendan Haigh, Vincent Perreten, Artur Summerfield

**Affiliations:** ^1^Institute of Virology and Immunology, Bern, Switzerland; ^2^AgResearch, Ruakura Research Centre, Hamilton, New Zealand; ^3^Vetsuisse Faculty, Department of Infectious Diseases and Pathobiology, Institute of Veterinary Bacteriology, University of Bern, Bern, Switzerland; ^4^Vetsuisse Faculty, Department of Infectious Diseases and Pathobiology, University of Bern, Bern, Switzerland

**Keywords:** cattle, Mx1, bacterial infection, nucleic acid sensing, type I IFN, cathelicidin, antimicrobial cationic peptides, epithelial cells

## Abstract

Cathelicidins encompass a family of cationic peptides characterized by antimicrobial activity and other functions, such as the ability to enhance the sensing of nucleic acids by the innate immune system. The present study aimed to investigate the ability of the bovine cathelicidins indolicidin, bactenecin (Bac)1, Bac5, bovine myeloid antimicrobial peptide (BMAP)-27, BMAP-28, and BMAP-34 to inhibit the growth of bacteria and to enhance the sensing of nucleic acid by the host’s immune system. BMAP-27 was the most effective at killing *Staphylococcus aureus, Streptococcus uberis*, and *Escherichia coli*, and this was dependent on its amphipathic structure and cationic charge. Although most cathelicidins possessed DNA complexing activity, only the alpha-helical BMAP cathelicidins and the cysteine-rich disulfide-bridged Bac1 were able to enhance the sensing of nucleic acids by primary epithelial cells. We also compared these responses with those mediated by neutrophils. Activation of neutrophils with phorbol myristate acetate resulted in degranulation and release of cathelicidins as well as bactericidal activity in the supernatants. However, only supernatants from unstimulated neutrophils were able to promote nucleic acid sensing in epithelial cells. Collectively, the present data support a role for certain bovine cathelicidins in helping the innate immune system to sense nucleic acids. The latter effect is observed at concentrations clearly below those required for direct antimicrobial functions. These findings are relevant in development of future strategies to promote protection at mucosal surfaces against pathogen invasion.

## Introduction

Antibiotics are widely used to treat infections in animals and to enhance growth and production. However, the spread of antibiotic resistance and the generation of multiresistant bacterial strains has become a worldwide problem, associated with the release of antibiotics in the environment (1). For these reasons, alternatives to antibiotics in controlling infectious disease are urgently required. One possible approach could be the stimulation of innate immune defense elements during critical phases of breeding.

Cathelicidins comprise of a heterogeneous class of host defense peptides (HDP) also termed as antimicrobial peptides. Neutrophils and epithelial cells mostly express these peptides in inactive forms that share an N-terminal signal sequence, a cathelin-like domain, and a highly diverse structural C-terminal part (2). The inactive pro-peptides are temporally stored within the specific granules of neutrophils. Upon activation, the C-terminal part of the molecule is cleaved from the cathelin-like domain by proteinases or elastases (3, 4). Interestingly, cattle possess all classes of cathelicidins recognized in higher vertebrates, whereas in humans only LL-37 was described. A total of seven cathelicidin peptides were detected in granules of bovine granulocytes including the cysteine-rich bactenecin (Bac)1, also known as dodecapeptide (5), the proline-rich peptides Bac5 and Bac7 (6), the tryptophan-rich indolicidin (7), and the α-helical bovine myeloid antimicrobial peptides (BMAP)-27, BMAP-28 (8), and BMAP-34 (9, 10). Although four putative cathelicidin pseudogenes were observed, no cDNA expression was detected (11). Henceforth, the seven protein-coding cathelicidin genes were annotated as *CATHL1* (Bac1), *CATHL2* (Bac5), *CATHL3* (Bac7), *CATHL4* (indolicidin), *CATHL5* (BMAP-28), *CATHL6* (BMAP-27), and *CATHL7* (BMAP-34) (12). *In situ* hybridization associated Bac5 to neutrophil infiltration under inflammatory conditions in pulmonary tissues, whereas its expression was absent in healthy lungs (13). Expression of *CATHL*s has been detected in a wide range of tissues including lungs, small and large intestines, mammary glands, and lymphoid tissues of cows (12). In addition, cathelicidins were found in milk from udders treated with LPS (14, 15) or following bacteria-induced mastitis (16–18), suggesting important roles of cathelicidins in fighting against invading pathogens.

In addition to their antimicrobial functions, we have recently shown that a prominent feature of porcine cathelicidins is their strong ability to enhance the sensing of nucleic acids by the innate immune system (19). Consequently, the potential protective role of bovine cathelicidins was investigated in this study. The aim was to evaluate their antimicrobial activity in relation to their ability to enhance nucleic acid-induced innate immune responses. Considering the importance of mastitis in dairy farming, three major mastitis pathogens, *Staphylococcus aureus, Streptococcus uberis*, and *Escherichia coli* were selected for our investigations.

## Materials and Methods

### Reagents

Bac1 (RLCRIVVIRVCR), Bac5 (RFRPPIRRPPIRPPFYPPFRPPIRPPIFPPIRPPFRPPLGPFP), indolicidin (ILPWKWPWWPWRR), BMAP-27 (GRFKRFRKKFKKLFKKLSPVIPLLHLG), sBMAP-27 (GLPKLHFRRKKGKLVSFPLKFKFKIRL), BMAP-28 (GGLRSLGRKILRAWKKYGPIIVPIIRIG), and BMAP-34 (AGLFRRLRDSIRRGQQKILEKARRIGERIKDIFRG) were synthesized using solid-phase peptide synthesis by ChinaPeptides (Shanghai, China). All peptides showed >98% purity by HPLC analysis and mass spectrometry. Peptides were dissolved in 2.5% DMSO (Bac1, Bac5, indolicidin, BMAP-28, and BMAP-34) or deionized sterile water (BMAP-27 and sBMAP-27) and stored at −80°C. Polyinosinic–polycytidylic acid [poly(I:C)], and phorbol myristate acetate (PMA) were purchased from InvivoGen (Toulouse, France). Bovine rIFN-α was purchased from Kingfisher Biotech Inc., Saint Paul, MN, USA. Detection of reactive oxygen species (ROS) was performed with the dye CM-H_2_DCFDA (Thermo Fisher Scientific, Switzerland). The integrity of the bacterial membrane was evaluated with BacLight™ Bacterial Membrane Potential Kit (Molecular Probes, Thermo Fisher Scientific).

Anti-human MxA (clone M143) was provided by Dr. J. Pavlovic (Institute of Medical Virology, University of Zürich, Zürich, Switzerland). Anti-mouse β-actin (clone AC-74) was obtained from Sigma-Aldrich (Buchs, Switzerland). Peroxidase-conjugated donkey anti-mouse IgG antibody was purchased from Jackson ImmunoResearch Labs (West Grove, PA, USA).

### Neutrophil Isolation and Stimulation

Bovine neutrophil granulocytes were isolated as previously described with a few adaptations (20, 21). Bovine blood sampling was performed in compliance with the Swiss animal protection law and approved by the animal welfare committee of the Canton of Bern, Switzerland, license number BE102/15. Briefly, 200 ml of blood was drawn from the jugular vein of cows at the Clinic for Ruminants (Vetsuisse Faculty, University of Bern, Switzerland) and was directly mixed with citrate-based anti-coagulant Alsever’s solution (1.55 mM of C_6_H_12_O_6_, 408 mM of Na_3_C_6_H_5_O_7_⋅2H_2_O, 1.078 mM of NaCl, and 43 mM of C_6_H_8_O_7_, pH 6.2). Afterward, the blood was centrifuged at 1,000 × *g* at 4°C for 20 min, the plasma and Buffy coat layer aspirated, and erythrocytes lysed in 12 ml ice cold hypotonic lysing solution (pH 7.3, 10.56 mM of Na_2_HPO_4_, and 2.67 mM of NaH_2_PO_4_ in sterile water). After 90 s, 6 ml of ice cold hypertonic solution (pH 7.3, 10.56 mM of Na_2_HPO_4_, 2.67 mM of NaH_2_PO_4_, and 0.43 M of NaCl in sterile water) was added to restore isotonic conditions. The cells were centrifuged at 800 × *g*, 4°C for 5 min and erythrocyte lysis repeated 2–3 times until red blood cells were no longer visible. Cells were then pooled into two Falcon tubes with 30 ml ice cold PBS/EDTA. A solution of 10 ml Ficoll-Paque 1.084 g/ml (GE Healthcare Europe GmbH, Freiburg, Germany) was overlaid with the cell suspension and centrifuged at 400 × *g* at room temperature for 40 min without brake. Neutrophils were recovered in the pellet, washed once with PBS/EDTA, counted in Trypan blue solution, and resuspended at 5 × 10^7^ cells/ml in RPMI 1640 medium (Biochrom GmbH, Berlin, Germany). For each animal, experiments were run in duplicate cultures. A total of 1 ml of 5 × 10^7^ neutrophils was seeded in a 24-well plate and were either stimulated with 100 ng/ml of PMA or kept unstimulated in medium for 10 min. Cells were cultured at 37°C in humidified 5% CO_2_ atmosphere. After 10 min, the plate was centrifuged at 400 × *g* and 4°C for 5 min. Supernatants were removed, and 1 ml medium was added to the cells. Neutrophils were incubated for a further 1 h at 37°C, before being centrifuged at 400 × *g* and 4°C for 5 min. Supernatants were collected and stored at −80°C until used. Aliquots of 5 × 10^6^ neutrophils per tube were used to quantify ROS. Briefly, neutrophils were incubated at 37°C with 5 µM of CM-H_2_DCFDA in 100 µl of HBSS (Thermo Fisher Scientific) for 15 min. Afterward, cells were washed and incubated further in fresh medium for 30 min. Cells were washed one more time, suspended in 200 µl of Cell Wash^®^ (Becton Dickinson, Basel, Switzerland), and acquired on a FACSCantoII (Becton Dickinson).

### Antimicrobial Assays

All bacterial strains including *S. aureus* strains (M3842, M905-1, and M3850) (22), *S. uberis* strains (M100/11, BL246, and ALP8092) (23) and *E. coli* (9217/10) were isolated from bovine mastitis milk (24). All strains were maintained on Trypticase soy agar plates with 5% sheep blood (Becton Dickinson AG, Allschwil, Switzerland) and always freshly plated the day before the experiment. Bacterial colonies were suspended in 0.85% NaCl solution (Axon Lab AG, Baden, Switzerland) to reach an OD value of 0.5 (DensiCHECK PLUS, Biomérieux, Genève, Switzerland), corresponding to approximately 1–1.5 × 10^8^ cell/ml. The minimum inhibitory concentration (MIC) of cathelicidins was determined by the broth microdilution method as previously described (25). Bacteria were further diluted to approximately 1 × 10^5^ cells/ml in cation-adjusted Mueller-Hinton (MH) broth (Thermo Fisher Scientific). Several twofold peptide dilutions (40–0.3 µM) were prepared in a volume of 50 µl MH II broth (Becton Dickinson AG) in a round-bottom 96-well plate. Finally, 50 µl of 10^5^ cells/ml were added to the peptide dilutions to reach a final concentration of 5 × 10^4^ cells/ml. The MIC was determined as lowest concentration without visible bacterial growth after overnight incubation at 37°C.

To determine the antimicrobial effects of BMAP-27 or sBMAP-27, bacterial suspensions of strains M3842, M100/11, and 9217/10 were incubated with several twofold dilutions of the peptides in a final volume of 100 µl of MH medium. Briefly, a volume of 50 µl containing 10^5^ cells/ml was mixed with 50 µl peptide dilution and incubated at 37°C for 1 h. As control, bacteria were incubated in the absence of cathelicidin. Each sample was then diluted 10-fold in PBS, and 50 µl suspension was plated on MH II agar petri dishes (Becton Dickinson). Colonies were counted after overnight incubation at 37°C and reported as CFU/ml. The bactericidal activity of neutrophil supernatants was evaluated using a similar protocol. Briefly, 95 µl of supernatants were pipetted in a flat-bottom 96-well plate and inoculated with 5 µl of 10^6^ cells/ml of *E. coli* suspension. After incubation at 37°C for 1 h, the samples were diluted 10-fold in PBS and plated on MH agar petri dishes. Colonies were enumerated after overnight incubation at 37°C and were reported as CFU/ml.

To evaluate bacterial membrane potential, approximately 10^7^ bacterial cells were suspended in 1 ml of filtered PBS solution and treated with BMAP-27 or sBMAP-27. Carbonyl cyanide 3-chlorophenylhydrazone (CCCP) was employed at 5 µM as positive control. Afterward, *E. coli* and *S. aureus* were treated with 30 µM, while *S. uberis* was stained with 60 µM of DiOC_2_(3). Cells were incubated 30 min at RT and acquired on a FACSCantoII. The gating strategy was defined to remove doublets and bacterial cell debris based on the different FSC and SSC parameters (Figure S1A in Supplementary Material). To evaluate the ability of cathelicidins to form pores in the membrane, around 10^7^ bacterial cells were suspended in 0.5 ml of filtered 0.85% NaCl solution in tubes and treated with BMAP-27 or sBMAP-27. As positive control, cells were incubated in 70% isopropanol. After 1 h incubation at RT, bacterial cell suspensions were centrifuged at 10,000 × *g* for 3 min and supernatants discarded. For staining of dead *E. coli*, 0.5 ml 7-aminoactinomycin D (7-AAD) at 1 µg/ml was used, while for *S. aureus* and *S. uberis* 0.5 ml of propidium iodide (PI) at 1 µg/ml was employed. Cells were incubated for 5 min at RT. Bacteria were further washed with 1 ml of 0.85% filtered NaCl, centrifuged at 10,000 × *g* for 3 min, and suspended in 0.5 ml of 0.85% filtered NaCl solution before being acquired on a FACSCantoII. Doublets, aggregates, and debris were excluded by electronic gating (Figure S1A in Supplementary Material).

### Gel Shift Assay

Cathelicidins were diluted in 8 µl of nuclease-free water at the indicated concentration and mixed with 2 µl of 100 ng pEAK8-His plasmid. The plasmid alone was employed as a control. Preparations were incubated at RT for 10 min. Subsequently, 2 µl of 6× loading dye (Thermo Fisher Scientific) were added. Finally, the mixtures were run on a 0.8% agarose gel and DNA visualized with ethidium bromide under UV radiation.

### Induction Mx and Western Blotting

Primary bovine turbinate epithelial cells (BT cells) were obtained as previously described (26) and the cultures maintained in DMEM supplemented with 10% FBS, non-essential amino acids (NEAA), neomycin/bacitracin, penicillin/streptomycin (stock solutions purchased from Biochrom), and GlutaMAX (Thermo Fisher Scientific). BT cells were plated in 24-well plates at 50,000 cells/well and incubated for 18 h at 37°C in 5% CO_2_. For induction of Mx, cathelicidins were mixed with 100 ng/ml of poly(I:C) in a final volume of 1 ml, incubated for 10 min at room temperature, and then added to the cells. Alternatively, different dilutions of neutrophil-derived supernatants were mixed with poly(I:C) at 100 ng/ml in complete medium (DMEM with 10% FBS, NEAA, neomycin/bacitracin, penicillin/streptomycin, and GlutaMAX). Positive controls for Mx1 induction included cell treatment with 100 ng/ml of bovine rIFN-α and 1 µg/ml of poly(I:C). Cultures were further incubated at 37°C for 20 h. After removal of the medium, the cells were lyzed by addition of 30 µl M-PER mammalian protein extraction reagent (Thermo Fisher Scientific) supplemented with complete protease inhibitor cocktail (Roche Diagnostics, Basel, Switzerland). Lysates were centrifuged at 10,000 × *g* at 4°C for 10 min and 20 µl supernatant collected for western blotting. Separation and blotting of proteins were performed as described earlier (26).

For western blots, 20 µl of primary epithelial cell lysates, obtained from approximately 5–10 × 10^4^ cells, were loaded and separated on 12% SDS-polyacrylamide gels (Bio-Rad, Reinach, Switzerland). Proteins were then blotted onto nitrocellulose membranes (Amersham Biosciences, Dübendorf, Switzerland) followed by a saturation step with PBS-T buffer containing 0.5% Tween 20 (v/v) supplemented with 5% (w/v) low-fat dry milk (Nestlé, Vevey, Switzerland). Afterward, membranes were incubated with the anti-human MxA and anti-mouse β-actin antibodies in PBS-T buffer supplemented with 0.5% milk (w/v) at 4°C overnight. The next day, a washing step with PBS-T was performed for 30 min. Membranes were further incubated with the peroxidase-conjugated donkey anti-mouse IgG antibody in 0.5% milk, 0.5% Tween20 PBS for 1 h. Subsequently, membranes were washed twice in 0.5% Tween 20 in PBS. Finally, solutions of WesternBright ECL HRP substrate (Advansta Inc., Menlo Park, CA, USA) were mixed in a 1:1 ratio and incubated with membranes for 2 min. Images were obtained using a CCD-LAS3000 camera (Fuji Film).

For the detection of cathelicidins in the neutrophil supernatants, a rabbit anti-cathelin domain polyclonal antibody (18) was employed at 10 µg/ml. For detection, a peroxidase-conjugated donkey anti-rabbit IgG was used as secondary antibody (Thermo Fisher Scientific). Neutrophil lysates were employed as a positive control.

### Software and Statistical Analysis

Helical wheel projection and net charge values were obtained using HeliQuest.^1^ Representation of BMAP-27 and sBMAP-27 were generated using I-TASSER (27, 28). The best model for BMAP-27 obtained a C-score of −0.42, while sBMAP-27 only reached −2.28. Peptide conformations were also predicted on PsiPred^2^ and Jpred.^3^ Analysis of flow cytometry files was undertaken using FlowJo v10. Data were analyzed and graphs generated using GraphPad Prism 6.0. Statistical analyses used matched paired *t*-test or multiple comparisons using one-way ANOVA. Dunnett’s multiple comparison *post hoc* test was employed to compare data with a control group. Significant differences are noted as *p < 0.05 or **p < 0.001.

## Results

### Conformation-Dependent Activity of BMAP-27 on Bacterial Membrane Integrity

**Figure 1:**
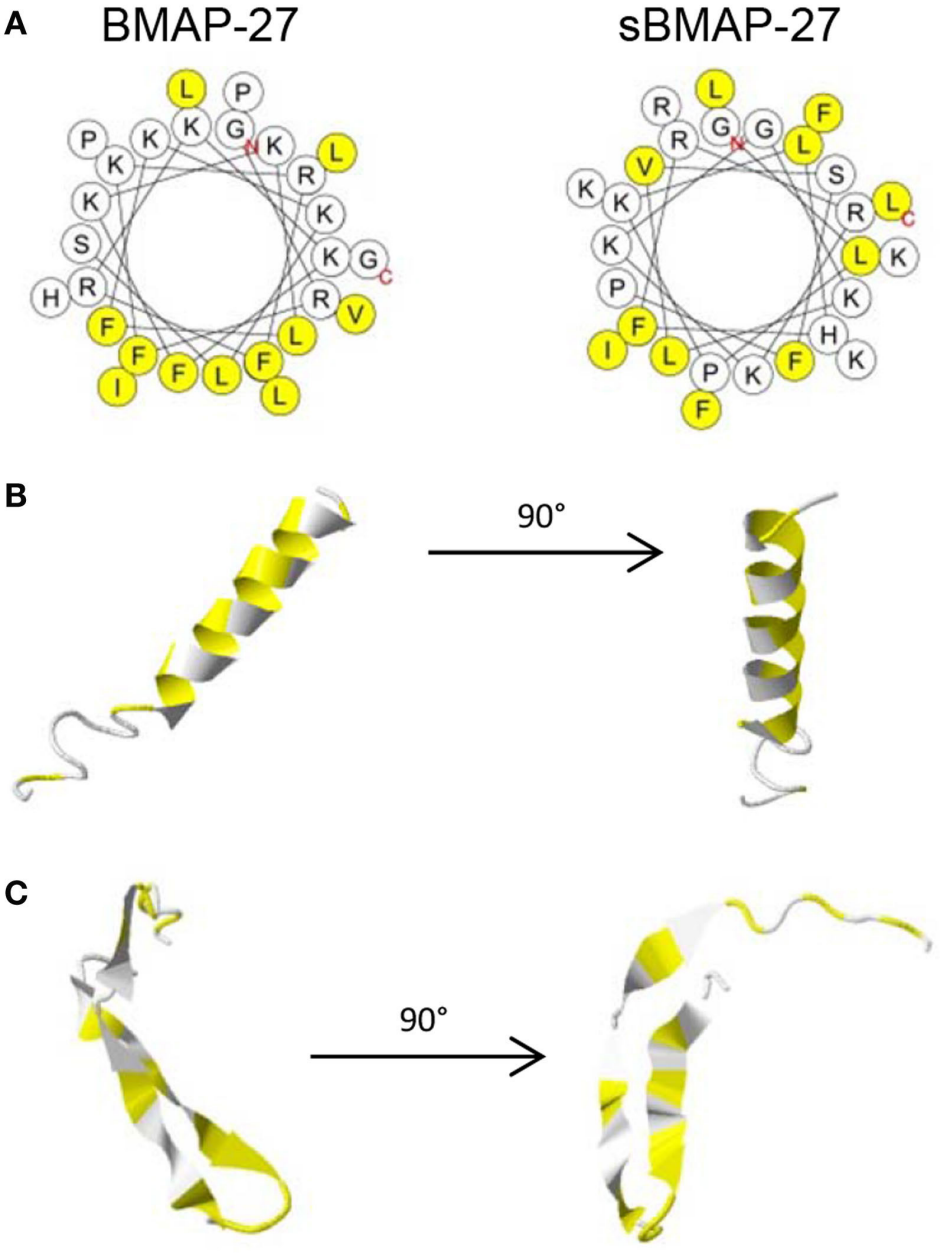
**Predicted conformation of bovine myeloid antimicrobial peptide (BMAP)-27 and sBMAP-27**. **(A)** Helical wheel projection of BMAP-27 and sBMAP-27. Hydrophobic residues are displayed in yellow, and the N- and C-terminal ends are depicted red. The helical projections were determined with HeliQuest. **(B,C)** Peptide ribbon of BMAP-27 **(B)** and sBMAP-27 **(C)** obtained by homology sequence comparison using I-Tasser. Hydrophobic portions are displayed in yellow. Arrows indicate a left side 90° rotation.

A combination of chemical factors including hydrophobicity, net charge, and the ability of HDP to form particular secondary structures functionally determines the antimicrobial activity of peptides. Leaving the net charge of +10 intact, we designed a scrambled version of BMAP-27 (sBMAP-27) to evaluate the impact of structure on biological activity. The amphipathic conformation characteristic of alpha-helical cathelicidins was made visible in a helical wheel projection (Figure 1A), as well as in a three-dimensional model of the original BMAP-27 (Figure 1B). This contrasted with sBMAP-27, which was predicted to form a beta-sheet structure (Figure 1C).

**Figure 2:**
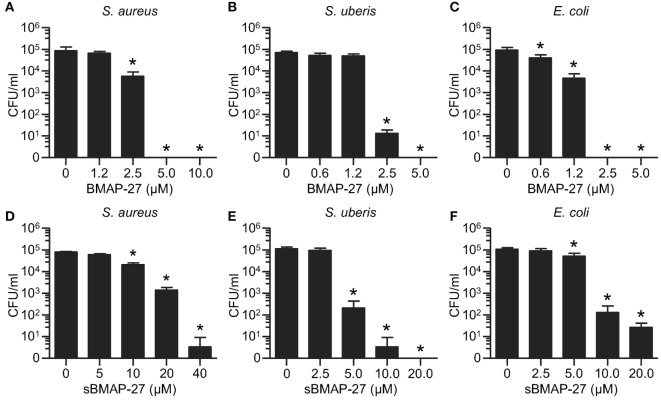
**Bactericidal properties of bovine myeloid antimicrobial peptide (BMAP)-27 and sBMAP-27 against mastitis isolates**. **(A–C)** BMAP-27 was mixed with *Staphylococcus aureus* M3842 **(A)**, *Streptococcus uberis* M100/11 **(B)**, and *Escherichia coli* 9217/10 **(C)** to reach a final bacterial concentration of 5 × 10^4^ CFU/ml and incubated at 37°C for 1 h. As control, bacteria were incubated in the absence of cathelicidin. Each sample was then diluted 10-fold in PBS and 50 µl of suspension plated on petri dishes. Colonies were counted and reported as CFU/ml. **(D–F)** Similar to BMAP-27, different dilutions of sBMAP-27 were mixed with *S. aureus* M3842 **(D)**, *S. uberis* M100/11 **(E)**, and *E. coli* 9217/10 **(F)**, incubated at 37°C for 1 h, plated and colonies enumerated. Significant differences compared to the controls were determined by Dunnett’s multiple comparison test (*p < 0.05).

**Figure 3:**
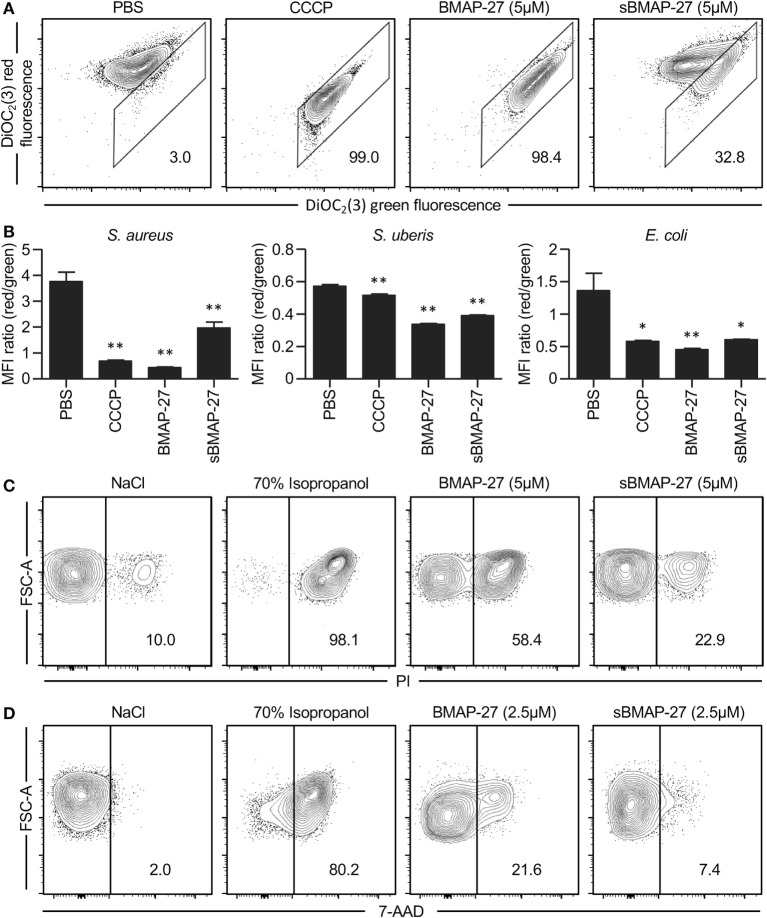
**Impact of bovine myeloid antimicrobial peptide (BMAP)-27 on bacterial membrane integrity**. **(A)** 10^7^ CFU of *Staphylococcus aureus* M3842 were incubated with 5 µM of BMAP-27 or sBMAP-27 together with the carbocyanine dye DiOC_2_(3) for 30 min to measure membrane potential. A shift from red to green fluorescence indicates a loss of membrane potential. The protonophore CCCP (5 µM) was employed as positive control. Plots are shown from a representative experiment out of three. **(B)** Ratio of red/green fluorescence determined for *S. aureus* M3842, *Streptococcus uberis* M100/11, *or Escherichia coli* 9217/10 incubated with BMAP-27 (5 µM), sBMAP-27 (5 µM), CCCP (5 µM), or PBS. Bar represents the mean fluorescence intensity ratio ± SD of triplicate cultures. Significant differences compared to the untreated control were determined by Dunnett’s multiple comparison tests (*p < 0.05; **p < 0.01). **(C)** *S. aureus* membrane permeabilization by BMAP-27. A total of 10^7^ CFU of *S. aureus* M3842 were incubated at the indicated concentration of cathelicidin for 1 h, washed, and then stained with propidium iodide (PI). Flow cytometry plots of one representative experiment out of two are presented and frequencies of PI-positive cells indicated. **(D)** *E. coli* membrane permeabilization by BMAP-27. A total of 10^7^ CFU of *E. coli* 9217/10 were incubated at the indicated concentrations of cathelicidin for 1 h and stained with 7-aminoactinomycin D (7-AAD). Plots of one representative experiment out of two are shown with frequency representing 7-AAD positive cells. Doublets and bacterial cell debris based on the different FSC and SSC parameters were excluded in all experiments.

As expected, sBMAP-27 had reduced antimicrobial activity when compared to BMAP-27. This was found with *S. aureus, S. uberis*, and *E. coli* and was in the range of a 4- to 10-fold reduction (Figure 2). These data suggest that the structural conformation of BMAP-27 mostly confers bactericidal function, although the role of the cationic charge cannot be excluded. Due to their amphipathic characteristics, cationic HDP typically affect the potential of bacterial membranes, which can be evaluated by flow cytometry using carbocyanine dyes (29). This test demonstrated that compared to sBMAP-27, BMAP-27 strongly affected membrane integrity of *S. aureus* (Figure 3A). Cathelicidin-induced changes of membrane potential were also observed with *S. uberis* and *E. coli*, but the impact of secondary structure was less evident (Figure 3B). In order to evaluate if BMAP-27 kills bacterial cells through the formation of pores (30), cell-impermeable nucleic acid stains were employed. For both Gram-positive bacteria, the frequency of PI-positive cells was always higher in the presence of BMAP-27 compared to its scrambled version (Figure 3C; Figure S1B in Supplementary Material). Similarly, *E. coli* treated with BMAP-27 showed a higher frequency of 7-AAD positive cells in comparison to sBMAP-27-treated *E. coli* (Figure 3D). Altogether, these data indicate that the bactericidal property of BMAP-27 is at least in part dependent on the amphipathic conformation, presumably impacting membrane integrity by forming pores into both Gram-negative and Gram-positive bacteria.

### Comparative Analysis of Antimicrobial Activity of Bovine Cathelicidins against Mastitis Pathogens

To evaluate the antimicrobial activity of bovine cathelicidins (5–10), six synthetic peptides including the alpha-helical BMAP-27, -28, and -34, the scrambled sBMAP-27, the tryptophan-rich indolicidin, the cysteine-rich disulfide-bridged peptide Bac1, and a proline-rich peptide Bac5, were employed to assess MIC against seven mastitis isolates (Table 1). Cathelicidins belonging to the alpha-helical peptide class were the most efficient in inhibiting bacterial growth. Surprisingly, indolicidin, Bac1 (dodecapeptide), and Bac5 had a low capacity to prevent the growth of all strains of *S. aureus* and *E. coli* at relevant *in vivo* concentrations (<20 µM). As reported above, 2- to 4-fold higher concentration of sBMAP-27 was required to reach MIC compared to BMAP-27, confirming the importance of the alpha-helical conformation to sustain optimal antimicrobial function.

**Table 1:** **Minimum inhibitory concentration (MIC) of six different bovine cathelicidins and a scrambled version of bovine myeloid antimicrobial peptide (BMAP)-27**.

	MIC (μM)						
	*Staphylococcus aureus*			*Streptococcus uberis*			*Escherichia coli*
Peptide	M3842	M3850	M905-1	M100/11	BL246	ALP8092	9217/10
BMAP-27	5.00 ± 0	7.22 ± 2.64	8.33 ± 2.5	2.78 ± 0.83	4.17 ± 1.25	4.72 ± 0.83	2.50 ± 0
sBMAP-27	≥20 ± 10	17.78 ± 4.41	20.00 ± 0	5.00 ± 0	5.56 ± 1.67	8.33 ± 2.5	10.00 ± 0
BMAP-28	13.33 ± 5	17.78 ± 4.41	14.44 ± 5.27	10.00 ± 0	10.00 ± 0	10.00 ± 0	10.00 ± 0
BMAP-34	12.22 ± 4.41	10.00 ± 0	14.44 ± 5.27	5.00 ± 0	4.17 ± 1.25	5.00 ± 0	5.00 ± 0
Indolicidin	>20	≥20 ± 6.67	>20	5.00 ± 0	7.22 ± 2.64	5.00 ± 0	>20
Bac1	>20	≥20 ± 6.67	>20	13.33 ± 5	20.00 ± 0	≥20 ± 10	>20
Bac5	>20	>20	>20	2.78 ± 0.83	6.11 ± 2.2	20.00 ± 0	>20

*Data represent the average ± SD of three independent experiments performed in triplicates*.

### Interaction of Bovine Cathelicidins with Plasmid DNA

**Figure 4:**
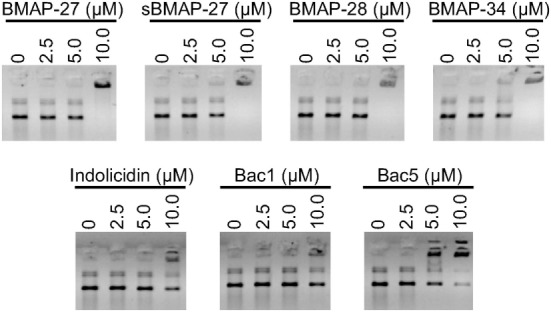
**Bovine cathelicidins interact with nucleic acids influencing plasmid migration**. Different dilutions of cathelicidins were incubated with 100 ng of DNA plasmid in nuclease-free water for 10 min, then loaded on a 0.8% agarose gel. Nucleic acids were visualized with ethidium bromide.

Previous studies demonstrated that many cationic HDP including cathelicidins (19, 31–33) and β-defensins (34) could interact with negatively charged nucleic acids. Consequently, we determined the ability of bovine HDP to influence nucleic acid migration in agarose gels. Migration patterns were strongly altered at 10 µM of BMAP-27, sBAMP-27, BMAP-28, BMAP-34, and Bac5 (Figure 4). These observations were confirmed by quantifying the signal of the lower band on the gels (Figure S2 in Supplementary Material).

### Ability of Bovine Cathelicidins to Promote Nucleic Acid Sensing by the Innate Immune System

**Figure 5:**
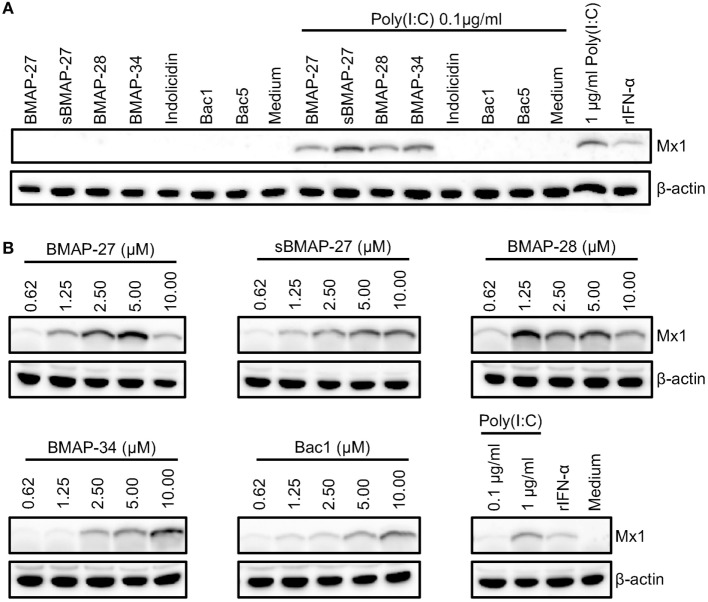
**Bovine myeloid antimicrobial peptide (BMAP)-27, sBAMP-27, BMAP-28, BMAP-34, and Bac1 promote nucleic acid sensing in primary epithelial cells**. **(A)** Bovine cathelicidins (5 µM) were complexed with 0.1 µg/ml poly(I:C) in medium at room temperature for 10 min before being added to bovine turbinate epithelial cells (BT cells). After 20 h incubation, Mx1 and β-actin were detected by western blot. One experiment out of three representative experiments is shown. **(B)** Cathelicidin dose-dependent induction of Mx1 in presence of poly(I:C). Increasing concentrations of bovine cathelicidins were mixed with 0.1 µg/ml of poly(I:C) in medium for 10 min and added to BT cells. After 20 h of culture, Mx1 and β-actin were detected by western blot. One representative experiment out of two is shown.

Both human and porcine cathelicidins have been described as forming complexes with free nucleic acids, favoring uptake by plasmacytoid dendritic cells (pDC), and leading to potent type I interferon (IFN) responses (19, 31). Considering that bovine pDC are very rare in peripheral blood (35), and that cathelicidins will also be secreted at mucosal surfaces, we employed a primary epithelial cell (BT cells) model and determined Mx1 induction as a readout for type I interferon responses (26). First, the titration of poly(I:C) promoting Mx1 expression was performed to evaluate the threshold at which these cells sensed poly(I:C). The induction of Mx1 was not detectable below a concentration of 156 ng/ml of poly(I:C) (Figure S3 in Supplementary Material). Consequently, 0.1 µg/ml was used to evaluate the ability of cathelicidins to promote nucleic acid sensing. Our results demonstrate that the addition of BMAP-27, sBAMP-27, BMAP-28, and BMAP-34 to poly(I:C) enabled the detection of Mx1, whereas this response was very weak or not observed with indolicidin, Bac1, or Bac5 (Figure 5A). Furthermore, a cathelicidin dose-dependent induction of Mx1 was shown with BMAP-27, sBAMP-27, BMAP-28, BMAP-34 as well as with the disulfide-bridged peptide Bac1 (Figure 5B). These data demonstrate that bovine alpha-helical and cysteine-rich disulfide-bridged cathelicidins can promote nucleic acid sensing at low peptide concentrations.

### Activated Neutrophils Release Cathelicidins but Cannot Induce Mx1 in Response to Poly(I:C)

**Figure 6:**
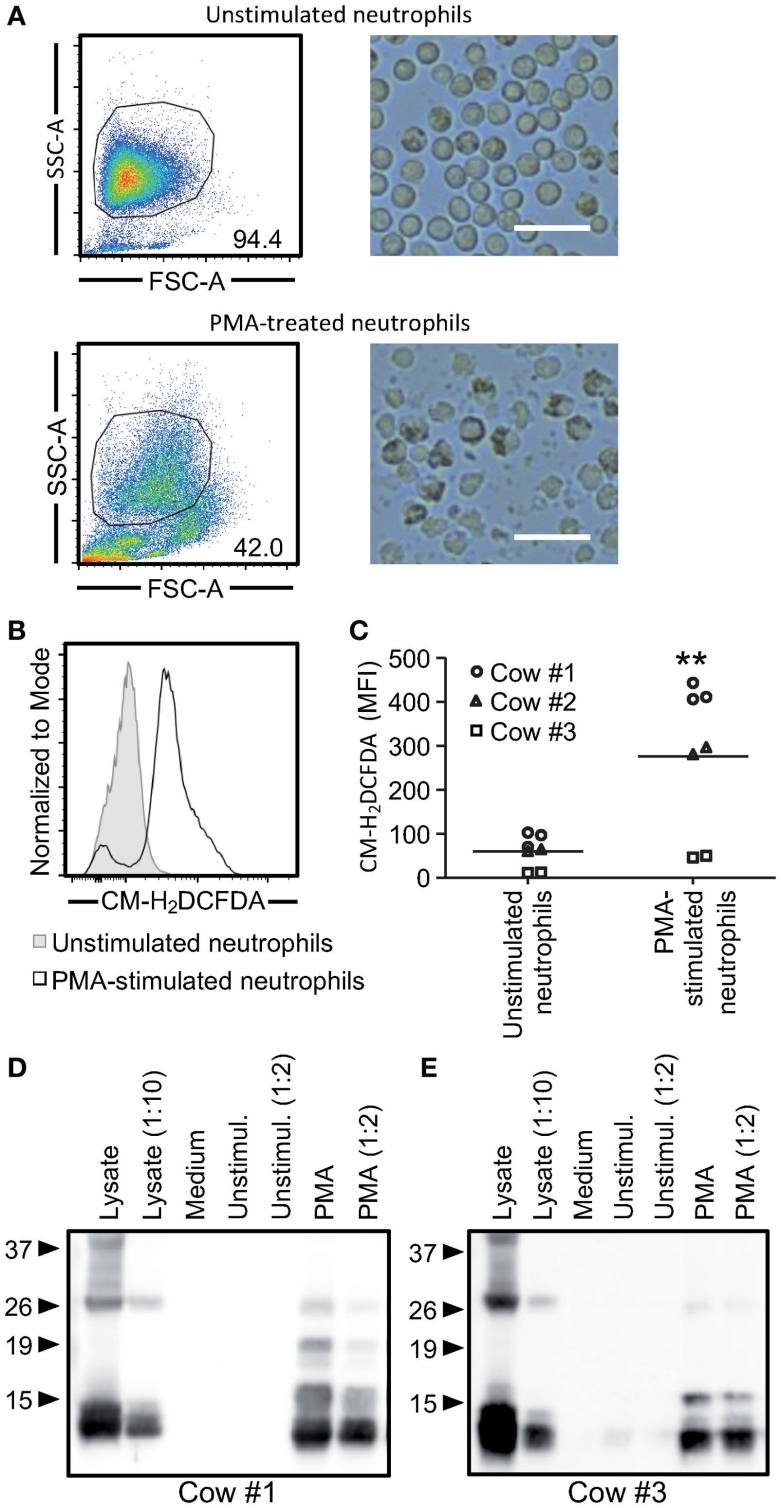
**Cathelicidins can be detected in the supernatants of phorbol myristate acetate (PMA)-activated neutrophils**. A total of 5 × 10^7^ neutrophils were stimulated with 100 ng/ml of PMA for 10 min, washed, and cultured for 1 h followed by analysis of reactive oxygen species (ROS) and cathelicidins. **(A)** Forward and side scatter characteristics of neutrophils stimulated with PMA or left unstimulated were evaluated by flow cytometry (left). Changes in morphology were observed by bright field microscopy with 100 magnification (right). White bars represent 25 µm. **(B)** Histogram overlay of CM-H_2_DCFDA fluorescence as an indicator of ROS formation. The unfilled histogram represents PMA-stimulated neutrophils and the gray-filled the unstimulated cells. Cow #1 is shown. **(C)** The graph shows mean fluorescence intensity of three independent animals each performed in duplicates. Each symbol represents an animal, and the line shows the average of all animals. Significant difference in the average was observed using a paired *t*-test (**p < 0.001). **(D,E)** PMA-stimulated neutrophils released cathelicidins into the medium. Undiluted and twofold diluted neutrophil supernatants were employed for western blots using a rabbit anti-bovine cathelin antibody (10 µg/ml). Cow #1 and cow #3 are shown on the left and right side, respectively. Neutrophil lysates were used as positive controls.

**Figure 7:**
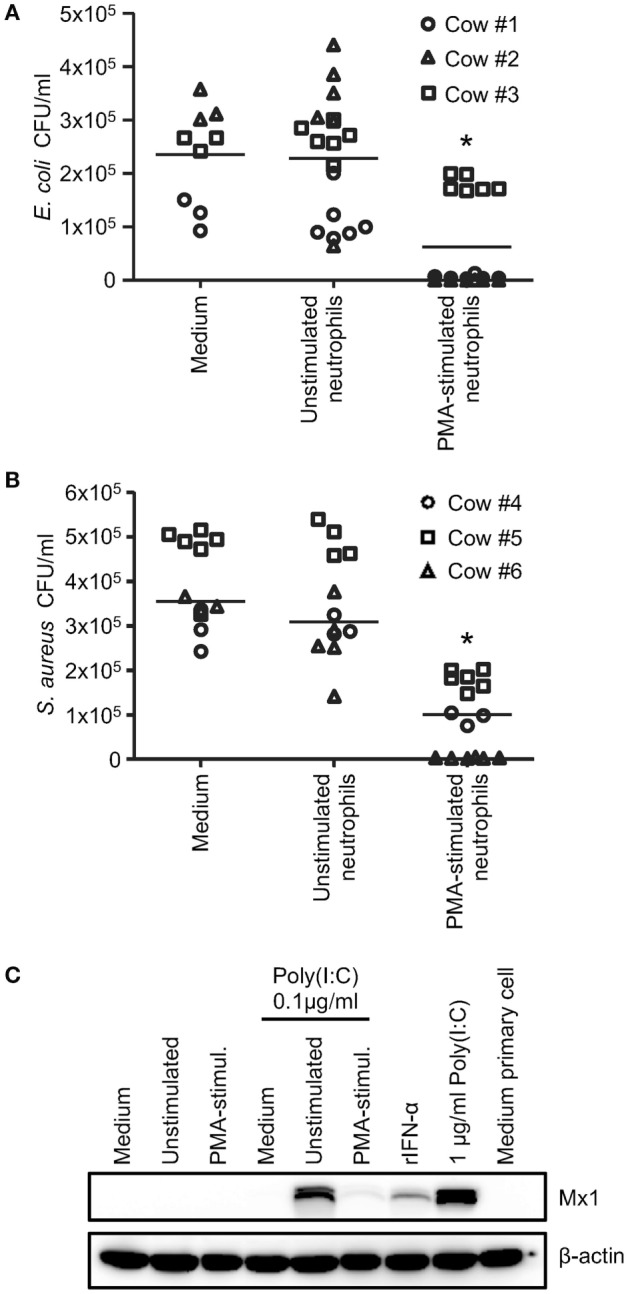
**Antimicrobial and Mx1-inducing activity of neutrophil-derived supernatants**. **(A,B)** Antimicrobial activity against both Gram-negative and Gram-positive bacteria was observed with supernatants of phorbol myristate acetate-treated neutrophils. Supernatants were mixed with approximately 5 × 10^3^ CFU of *Escherichia coli* 9217/10 **(A)** and *Staphylococcus aureus* M3842 **(B)**, and incubated at 37°C for 1 h. As control, bacteria were incubated in FBS-free medium. Each sample was then diluted 10-fold in PBS, and 50 µl of suspensions were plated on petri dishes to determine the CFU/ml. Each symbol represents at least a triplicate from one animal, and the line shows the average of all animals. Significant differences compared to the medium alone were calculated using Dunnett’s multiple comparison test (*p < 0.05). **(C)** Enhancement of nucleic acid sensing in bovine turbinate epithelial cells (BT cells) by neutrophil supernatants. Supernatants were incubated with or without 0.1 µg/ml poly(I:C) for 10 min before being transferred to BT cells. After 20 h, Mx1 and β-actin expression were determined by western blot. One representative experiment out of four is shown.

In order to clarify the importance and relevance of neutrophil-derived cathelicidins in mediating these responses, bovine blood neutrophils were used as a source of cathelicidins. To induce neutrophil activation, we employed PMA stimulation. The observed reduction in side scatter determined by flow cytometry, as well as the morphological changes observed by bright field microscopy (Figure 6A) indicated neutrophil degranulation following PMA stimulation. Activation was also confirmed by the quantification of ROS by flow cytometry (Figure 6B). Although some variations were noticed between animals, the fluorescence intensity of CM-H_2_DCFDA (ROS indicator) of PMA-stimulated neutrophils was significantly higher compared to unstimulated neutrophils (Figure 6C). As expected, the activation of PMA-treated neutrophils was also characterized by the release of cathelicidins detected by western blot (Figures 6D,E). To evaluate antimicrobial function, neutrophil supernatants were tested for their ability to reduce the growth of *E. coli* and *S. aureus*. No significant effects were found with supernatants of unstimulated neutrophils when compared to medium with both *E. coli* and *S. aureus*. However, 3.7-fold *E. coli* and a 4-fold *S. aureus* CFU reduction was observed with supernatants from PMA-stimulated neutrophils compared to medium (Figures 7A,B). Finally, we investigated the ability of neutrophil supernatants to promote nucleic acid sensing. Surprisingly, supernatants from unstimulated neutrophils incubated with poly(I:C) promoted Mx1 expression, whereas PMA-activated neutrophil supernatants elicited poor Mx1 expression in response to poly(I:C). In the absence of poly(I:C), no induction could be reported (Figure 7C).

## Discussion

This study was initiated to characterize bovine cathelicidins with respect to their capacity to mediate antibacterial and immunostimulatory responses at mucosal surfaces. Stimulating such activity has the potential to enhance resistance of cattle to infection. Considering the importance of mastitis for dairy cow health and that bovine cathelicidin gene expression has been reported in mammary tissue (12), we selected three major bacterial bovine mastitis pathogens for our investigations. We found that BMAP-27 strongly affects bacterial membrane integrity and probably forms pores in both Gram-positive and Gram-negative mastitis pathogens. It was previously shown that the 18 N-terminal residues of BMAP-27 were highly effective at inhibiting growth of many bacterial strains (36). Similar to LL-37 (37), the pore-forming capacity and antimicrobial mechanism of BMAP-27 was related to the amphipathic alpha-helical structure leading to membrane disruption (37). Like all cationic HDP, the mechanism is initiated by the electrostatic interactions of the HDP and negatively charged residues of the bacterial cytoplasmic membrane. In the case of α-helical cathelicidins, this step is followed by toroidal pore formation resulting in bacterial cell death (38). These findings contrast with the mode of action of other classes of cathelicidins such as tryptophan-rich peptide, indolicidin, or proline-rich peptide, Bac7, which inhibit bacterial DNA synthesis (39, 40) or RNA translation in *E. coli* (41), respectively. Surprisingly, sBMAP-27 still demonstrated antimicrobial function despite a complete loss of the amphipathic conformation, suggesting that the net charge of HDP contributes partly to the bactericidal effects. Recently, BMAP-27, BMAP-28, Bac5, and indolicidin were reported to share broad-spectrum antibacterial activity against several mastitis pathogens (42). The MIC values obtained with the present study were slightly higher compared to previous reports with the same species (5–10). This might be attributed to differences in the protocol employed, such as the preparation of the bacterial inoculum or the presence of different salt concentration, which can strongly impact peptide function.

Although many reports have aimed to define and characterize the antimicrobial activity of cathelicidins against various pathogens, very little is known regarding the immunomodulatory effects elicited by these peptides. Tomasinsig et al. demonstrated that both BMAP-27 and BMAP-28 promoted the release of TNF in a bovine mammary cell line (42). In the present study, it is shown that the sensing of double-stranded RNA analog was improved when bovine cathelicidins were added to nucleic acids prior to exposure to epithelial cells. As a readout, we employed the induction of Mx1, which is dependent on the induction of type I IFN (43). In both human and porcine models, it was proposed that pDC play a pivotal role in sensing of nucleic acids complexed to cathelicidin followed by the release of large quantities type I IFNs (19, 31). Here, we demonstrate that type I IFN can also be induced in epithelial cells. This could be immunologically relevant considering the role of these cells as a first defense against infection. Interestingly, the induction of Mx1 was also observed with the cysteine-rich Bac1 (dodecapeptide). Similarly, type I IFN was detected when β-defensins or protegrin were employed in the presence of nucleic acids in both human and porcine pDC (19, 34), respectively. Cysteine-rich cathelicidins, which structurally resemble defensins, may display conserved modulatory functions such as the ability to complex microbial DNA or RNA and promote the recognition by innate immunity. Cathelicidin-complexed nucleic acids could trigger cytoplasmic RNA or DNA receptors, or alternatively endosomal toll-like receptors. Further research is required to determine in which compartment the nucleic acid is delivered.

Type I IFNs not only mediate antiviral protection but also have a potent impact on adaptive immune responses by promoting both cellular immunity and antibody responses (44). Nevertheless, it is still controversial if type I IFNs have a beneficial or negative effect on bacterial disease outcome, and this appears to be pathogen dependent. For example, *in vivo* studies in mice indicate that *S. aureus*-triggered type I IFNs can be detrimental (45). On the other hand, type I IFN signaling is required to control disease and increase survival rate after other pathogenic bacterial infections, including Group A streptococci (46), Group B streptococci, pneumococci, *E. coli* (47), and *Streptococcus pyogenes* (48). In cattle, a single study reported that *S. uberis* could upregulate the transcription of IFN-ω (49). However, the link between IFN induction and protective immunity during bovine mastitis and bacterial infections in general has never been evaluated.

The induction of Mx1 was only detected with non-activated neutrophils, while the opposite was found with respect to the antimicrobial activity, requiring stimulation with PMA. A possible explanation is that the nucleic acid complexing activity requires lower doses, which are already released from neutrophils following their isolation and culture. In contrast, PMA induces a very strong neutrophil degranulation, releasing a multitude of components, some of which might have inhibitory activity on the process of Mx1 induction. We also know from our previous work that high concentrations of cathelicidins have inhibitory activity on IFN type I responses, presumably through cytotoxic effects (19). Alternatively, HDP have been reported to possess regulatory activity. Brook et al. demonstrated that the α-defensins secreted by neutrophils can inhibit TNF-α mRNA translation in macrophages (50). This observation is in accordance with the fact that many cathelicidins can neutralize endotoxins (51, 52), limiting an excessive inflammation. It thus appears that the composition and concentration of HDP present in the microenvironment can determine the type of immune response.

In conclusion, the present data provide a comprehensive overview of the antimicrobial functions of some bovine cathelicidins against mastitis pathogens, demonstrating cathelicidin- and bacteria-specific differences. We show that some of these peptides participate in the sensing of nucleic acids mediating IFN type I responses in epithelial cells. This work will help to identify ways of exploiting the biological effects of cathelicidins, to identify alternatives for antibiotics as has been proposed by others (53). In the case of bovine mastitis, it would be interesting to evaluate the exact contribution of these peptides during the course of the disease. The observations that HDP are a highly abundant in mastitis milk (16, 18) and also that the process of NETosis involving cathelicidins was found during mastitis (54) would support the maintenance of HDP’s biological activities in the udder. Furthermore, their exploitation for other mucosal surfaces such as the respiratory tract is attractive.

## Ethics Statement

Bovine blood sampling was approved by cantonal authorities (license BE102/15). All animal owners were asked for approval before blood drawing. Cows were patients from the Clinics for Ruminants (Vetsuisse Faculty, University of Bern, Switzerland).

## Author Contributions

MK, AB, and AS designed and performed experiments and wrote the manuscript. BH and VP provided essential reagents, advice, and scientific input.

## Conflict of Interest Statement

The authors declare that the research was conducted in the absence of any commercial or financial relationships that could be construed as a potential conflict of interest.

## Footnotes

^1^http://heliquest.ipmc.cnrs.fr/.

^2^http://bioinf.cs.ucl.ac.uk/psipred/.

^3^http://www.compbio.dundee.ac.uk/www-jpred/.

## Abbreviations

7-AAD, 7-aminoactinomycin D; Bac, bactenecin; BMAP, bovine myeloid antimicrobial peptide; HDP, host defense peptides; IFN, interferon; pDC, plasmacytoid dendritic cells; MH, Mueller-Hinton; NEAA, non-essential amino acids; PI, propidium iodide; PMA, phorbol myristate acetate; poly(I:C), polyinosinic–polycytidylic acid; ROS, reactive oxygen species.
