# Supplementary material for: Differential Ability of Bovine Antimicrobial Cathelicidins to Mediate Nucleic Acid Sensing by Epithelial Cells
Source: Front Immunol. 2017 Feb 1;8:59. doi: 10.3389/fimmu.2017.00059 (PMC5285380; doi:10.3389/fimmu.2017.00059)
Supplement: Supplementary file 1 [file image_1.pdf]

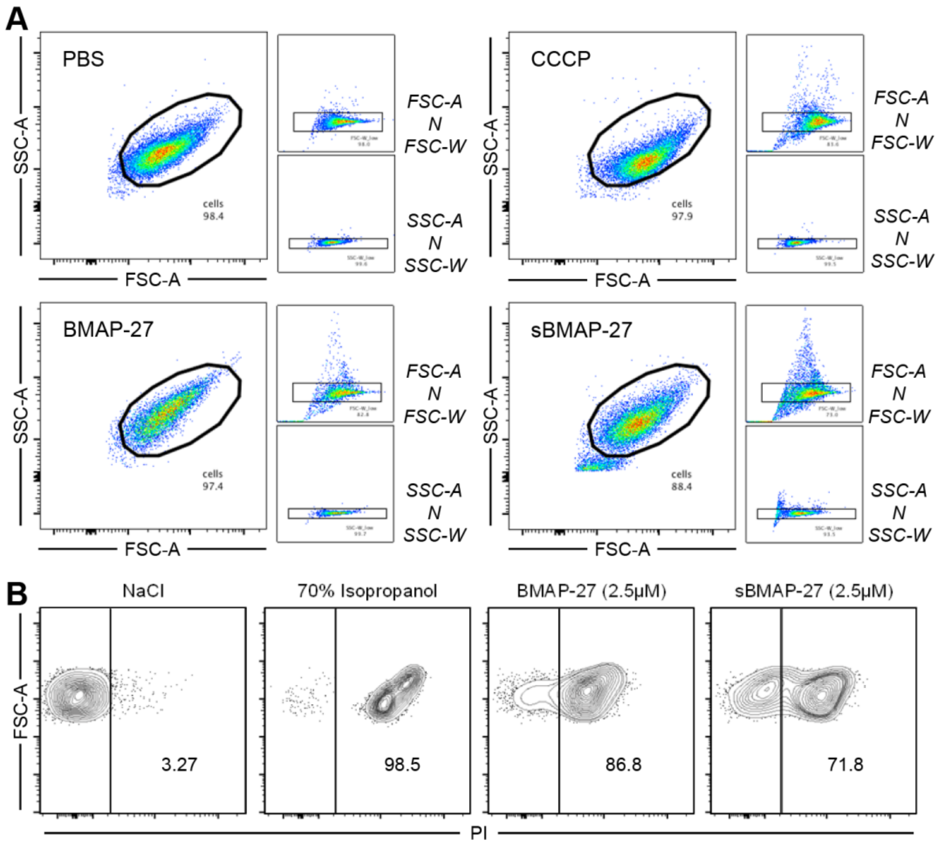

Supplemental figure 1

Flow cytometry analysis of bacterial membrane permeabilization by BMAP-27. (A) Gating strategy employed to remove debris and doublets. Forward and side scatter characteristics of *S.aureus* after 30 min of treatment with PBS, CCCP (5 μM), BMAP-27 (2.5μM) or sBMAP-27 (2.5μM). (B) BMAP-27 induced bacterial membrane permeabilization. Approximatively 10^7 CFU of *S.uberis* M100/11 were incubated at the indicated concentration of cathelicidin for 1h, washed and were then stained with PI. Representative dot plot of one experiment out two is depicted with frequency of PI positive cells indicating membrane disruption.

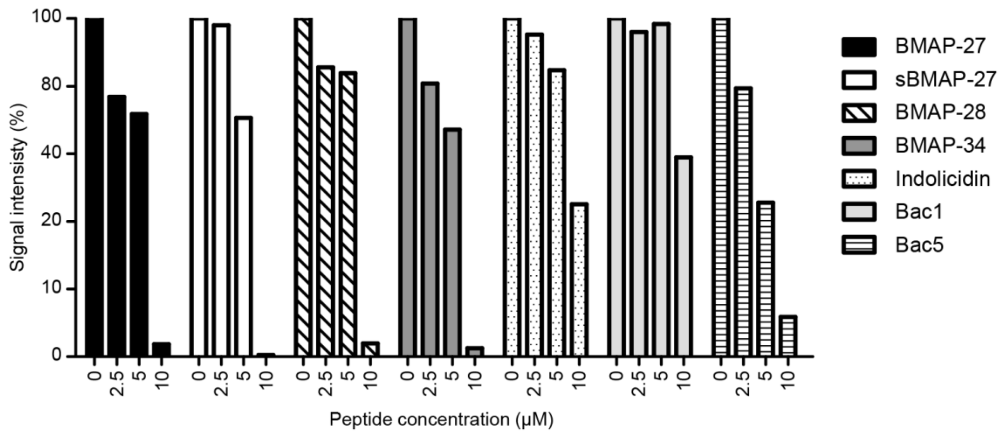

Supplemental figure 2.

Bovine cathelicidins interact with nucleic acids influencing DNA plasmid migration. Serial 2-fold cathelicidin dilutions were incubated with 100 ng of plasmid in nuclease-free water at RT for 10 min. Mixtures were loaded on 0.8 % agarose gel and nucleic acids were stained with ethidium bromide. Pictures were taken under UV exposure. Signal quantification was quantified from the faster band after plasmid migration with Aida Image Analyzer v.4 software.

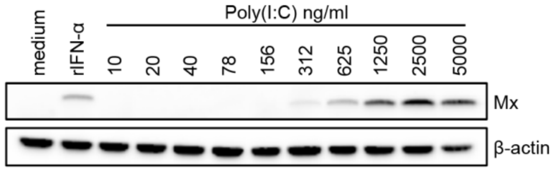

Supplemental figure 3. Titration of poly(I:C) for Mx1 induction in BT cells. After 20h incubation, cells were lysed and employed for the detection of Mx1 and β-actin by western blot.
